# Health Insurance, Socio-Economic Position and Racial Disparities in Preventive Dental Visits in South Africa 

**DOI:** 10.3390/ijerph10010178

**Published:** 2012-12-31

**Authors:** Imade J. Ayo-Yusuf, Olalekan A. Ayo-Yusuf, Bukola G. Olutola

**Affiliations:** 1 Department of Dental Management Sciences, School of Dentistry, Oral & Dental Hospital, Faculty of Health Sciences, University of Pretoria, Pretoria 0001, South Africa; E-Mail: imade.ayoyusuf@up.ac.za; 2 Department of Community Dentistry, School of Dentistry, Oral & Dental Hospital, Faculty of Health Sciences, University of Pretoria, Pretoria 0001, South Africa, E-Mail: bukola.olutola@gmail.com

**Keywords:** dental services utilization, health insurance, social gradient, disparities, race

## Abstract

This study sought to determine the contributions of socio-economic position and health insurance enrollment in explaining racial disparities in preventive dental visits (PDVs) among South Africans. Data on the dentate adult population participating in the last South African Demographic and Health Survey conducted during 2003–2004 (n = 6,312) was used. *Main outcome measure*: Reporting making routine yearly PDVs as a preventive measure. Education, material wealth index and nutritional status indicated socio-economic position. Multi-level logistic regression analysis was conducted to determine the predictors of PDVs. A variant of Blinder-Oaxaca decomposition analysis was also conducted. Health insurance coverage was most common among Whites (70%) and least common among black Africans (10.1%) in South Africa. Similarly, a yearly PDV was most frequently reported by Whites (27.8%) and least frequently reported among black Africans (3.1%). Lower education and lower material wealth were associated with lower odds of making PDVs. There was significant interaction between location (urban/rural) and education (p = 0.010). The racial and socio-economic differences in PDVs observed in urban areas were not observed in rural areas. In the general dentate population, having health insurance significantly increased the odds of making PDVs (OR = 4.32; 3.04–6.14) and accounted for 40.3% of the White/non-White gap in the probability of making PDVs. Overall, socio-economic position and health insurance enrollments together accounted for 55.9% (95% CI = 44.9–67.8) of the White/non-White gap in PDVs. Interventions directed at improving both socio-economic position and insurance coverage of non-White South Africans are likely to significantly reduce racial disparities in PDVs.

## 1. Introduction

South Africa is a middle-income country with a population of 46.9 million people [1], and with a history of massive social and economic inequalities resulting from 45 years of apartheid, which was formally abolished in 1994 [2]. A reasonably well-established public health system co-exists with a private health sector. Wide disparities in health spending, professional staffing levels and accessibility continue to exist between the public and private health sectors, amid escalating health care costs [3]. As there are currently no publicly-funded health insurance schemes, the main criterion for access to health insurance and thus to private health care in South Africa is formal employment [4]. The historically disadvantaged black Africans, who are still more likely than any other race group to be unemployed, continue to be less likely to be insured than Whites in South Africa [5]. Employers contribute up to two-thirds of an employee’s total monthly health insurance premium as part of a tax deductible benefit [6]. There are no stand-alone dental insurance plans, but most of the health insurance plans include dental benefits [7], to a varying extent. However, a visit to a dentist at least once a year for preventive dental care such as dental prophylaxis is covered in most South African health insurance plans and has been recently recommended by the Council for Medical Schemes to be included as part of basic dentistry to be covered under the ‘prescribed minimum benefit’ package, which is recognized by statute in South Africa [8]. 

The use of health services is a function of several factors, which include socio-demographic characteristics such as age, gender, and ethnicity. Other factors include the individual’s means of obtaining the healthcare he or she requires (such as his or her income level and/or being in possession of health insurance), and the perceived need [9]. Wang and others have demonstrated significantly lowered chances of experiencing a financial barrier to accessing the necessary dental care for children from a low-income family after the implementation of health insurance coverage for eligible children in a US population [10]. Other studies, mainly from developed countries, have also argued that providing universal insurance coverage increases health service utilization [11,12]. Similarly, it has been suggested that since the introduction of “free” primary oral health care in South Africa, the number of dental visits increased by 71% between 1995 and 2002, although such visits are still mainly made to obtain relief from pain and sepsis (symptomatic visits) [13]. 

However, some have suggested that providing universal insurance coverage may not increase dental utilization [14], and that, even if it did, it may not eliminate disparities in health care utilization [15,16]. Considering that addressing social disparities in the use of health services is one of the major justifications for the proposal to introduce National Health Insurance (NHI) in South Africa and that there is only limited empirical evidence of the role that health insurance plays in dental service utilization in South Africa, it is important to evaluate the potential role of health insurance in reducing (if not eliminating) racial disparities in access to preventive dental care in South Africa. Given that a visit to a dental office at least once a year for a check-up and routine professional cleaning has been widely recommended as an effective way to promote oral health [17], it was the aim of this study to explore socio-economic and racial disparities in preventive dental care utilization, and to quantify the contribution of having a health insurance and the observed racial socio-economic differences in explaining racial disparities in preventive dental visits (PDVs).

## 2. Methods

### 2.1. Data Source and Study Design

Data for this study were obtained from the last South African Demographic and Health Survey (SADHS), which is the most recent and largest nationally representative health survey that is publicly available in South Africa. This study involved individuals aged ≥15 years (n = 8,115) who participated in the SADHS conducted between October 2003 and August 2004. The details of the sampling procedure used in the SADHS have been previously published [18]. Briefly, the SADHS was a nationally representative, cross-sectional household survey, which used a stratified, two-staged probability sample design. The first stage involved selecting census enumeration areas (EAs) as the primary sampling units, with a probability proportional to size, based on the number of households in the EAs. The second stage involved a systematic sampling of households from the selected EAs. The data consisted of ten strata, one for each of the nine provinces, with 1,000 households allocated to each stratum. An additional stratum was selected in order to cover sample areas with Indian/Asian households, because of the small percentage (≤3%) of this group in the South African population. For the purposes of the current study, only dentate participants were included (n = 6,312).

### 2.2. Data Collection Procedure and Measures

Trained fieldworkers administered the questionnaires, which were prepared in all of South Africa’s 11 official languages.

### 2.3. Measures

#### 2.3.1. Socio-Demographic Characteristics

The SADHS used an interviewer-administered questionnaire to obtain the demographic characteristics of the population, including information on age, gender, race and education. Participants were asked what the highest level of school they had completed was. Based on the responses in years, respondents were categorized into three groups, namely “<high school”/12 years of schooling, “high school”/12 years of schooling and “>high school”/12 years of schooling. 

#### 2.3.2. Material Wealth Index

Consistent with the literature that suggests using multiple measures to capture indicators of socio-economic position along a person’s life course [19], a material wealth index was measured using the question “Does your household have any of the following items in working condition—a radio?, television (TV)?, computer?, refrigerator?, landline telephone?, a cell phone?” The respondents were also asked if any member of the household had a bicycle, a motorcycle or motor scooter, or a car or truck. Based on principal components analysis, the best fitted items were found to be a car, radio, TV, computer, refrigerator, landline and cell phone. The reliability coefficient for this 7-item scale was good (Cronbach α = 0.78). The index scores derived from adding up the response options “No” (coded 0) or “Yes” (coded 1) were then ranked to classify the study participants into three categories, namely, the lowest, middle and highest material wealth index tertiles. 

#### 2.3.3. Household Member Per Room (Crowding)

The number of household members per room was one of proxy used for socio-economic position, as there was limited information on household income, due to many missing income data. Household crowding was measured by dividing the total number of household members by the number of rooms in the house [19].

#### 2.3.4. Nutritional Status (Food Security)

Considering a previous report that low levels of food security can compete with dental care utilization in disadvantaged people [20], we also obtained information on participants’ nutritional status as a proxy measure for their level of food security. Nutrient intake was assessed by means of a 30-item food frequency questionnaire as part of the Nutritional Index (N-Index) developed for South Africa [21]. The maximum micronutrient score obtainable was 45 points—the higher the score, the worse the person’s nutritional status [22]. The total scores were then auto-ranked in order to categorize the study participants’ food nutrient levels into three categories, namely those in the lowest (poorest), middle and highest tertiles of nutritional status. 

#### 2.3.5. Tobacco Use Status

Given the long-lasting effect of tobacco use on periodontal health and thus a predisposition for needing regular dental care, and the previously reported association between tobacco use and dental care utilization [23], we documented participants’ “ever use” of tobacco. Specifically, those who responded in the affirmative to the question on any current or past use of either smokeless (snuff) or smoked tobacco products were classified as ‘ever snuff users’ or “ever smokers” respectively. 

#### 2.3.6. Self-Reported Dental Problems

Considering that the experience of a dental problem in the recent past may be associated with a need for dental service, including preventive dental care, we recorded any recent dental problem as perceived by the participants. The survey participants were asked the question ‘Have you had pain or problems with your mouth and/or teeth in the last six months?’ These respondents were then asked to indicate which part of the mouth was affected and the options “teeth” and “gums” were given. Respondents categorised as having dental problems were those who responded in the affirmative to having experienced teeth and/or gum problems. 

#### 2.3.7. Health Insurance Status

Participants were asked whether they were covered by a medical aid or medical scheme (health insurance). Those who responded in the affirmative were categorized as being “privately insured”.

#### 2.3.8. Preventive Dental Service Utilization

Participants were also asked what they usually did to look after their teeth/mouth. The options given (multiple responses were allowed) were “do nothing”, “clean/brush/floss” and “visit the dentist/dental therapist/oral hygienist/oral therapist at least once a year”. Anyone who indicated visiting a dental practitioner at least once a year was categorized as making a yearly preventive dental visit (PDV).

### 2.4. Data Analysis

All statistical analyses were done using STATA version 10 (Stata Corp, College Station, TX, USA), adjusting for the complex sample design by using the “svy” command option. Differences in weighted proportions and means were tested using the Chi-square and t-tests respectively. 

A multi-level logistic regression model was constructed in order to adjust for clustering at the primary sampling unit, *i.e.*, the EAs, and to provide robust standard error estimates [24]. Considering that access to dental care may differ by location, given that there are much fewer dental professionals working in the rural areas than in the urban areas of South Africa [25], we explored the role of residential location and health insurance as moderators of making PDVs. We tested for the interaction between location and race, location and education, health insurance and race, and race and education. The main outcome variable was yearly PDVs. 

To explore further the extent to which racial differences in socio-economic characteristics and insurance enrolment explain racial differences in PDVs, we used a variant of Blinder-Oaxaca decomposition [26]. This enabled us quantify the extent to which racial differences in the observed distribution of the characteristics explain racial disparities in terms of PDVs (*i.e.*, the indirect effects of race). 

### 2.5. Results

Of the dentate population studied, 57.5% (n = 3,707) were females and 83.8% (n = 4662) identified themselves as black Africans. Yearly PDVs were reported by 4.9% (n = 270), while 15.4% (n = 911) reported having medical aid/health insurance.

Respondents who lived in more crowded households were significantly less likely to be insured and were less likely to report a yearly PDV (p < 0.05). Those who had less than 12 years of schooling (high school) were also less likely to be privately insured and less likely to report a yearly PDV (Table 1). Health insurance coverage was most common among Whites (70.0%) and least common among black Africans (10.1%) in South Africa. Similarly, a yearly PDV was most frequently reported by Whites (27.8%) and least reported among black Africans (3.1%). Those who had the poorest nutritional status, those who live in rural areas and those who reported having had a dental problem in the recent past were also less likely to report making a yearly PDV (Table 1).

**Table 1:** Bivariate association between socio-demographics, health insurance coverage and preventive dental visits.

		% Privately Insured (n)	*p-value*		% visiting yearly (n)	*p-value*
Ethnicity/Race			<0.001			<0.001
	Black African	10.1 (427)			3.1 (123)	
	Coloured	21.6 (126)			3.2 (19)	
	Indian/Asian	32.9 (195)			10.5 (54)	
	White	70.0 (163)			27.8 (74)	
Location			<0.001			0.001
	Urban	19.9 (744)			5.7 (208)	
	Rural	6.2 (167)			3.2 (62)	
Education			<0.001			<0.001
	<High school	8.3 (400)			2.3 (100)	
	High School	23.5 (248)			7.9 (92)	
	>High School	56.5 (263)			19.2 (78)	
Gender			0.958			0.303
	Male	15.4 (383)			4.5 (109)	
	Female	15.4 (528)			5.2 (161)	
Nutritional status			<0.001			<0.001
	Poorest	7.4 (262)			2.2 (60)	
	Middle	20.8 (402)			6.7 (122)	
	Highest	19.9 (247)			6.4 (88)	
Tobacco use			<0.001			0.002
	Never	16.2 (650)			5.4 (204)	
	Ever snuff user	4.4 (17)			0.7 (4)	
	Ever smoker	16.2 (244)			4.5 (62)	
Dental problem			0.400			0.009
	No	15.6 (966)			5.2 (235)	
	Yes	14.2 (145)			2.8 (35)	
Privately insured						<0.001
	No	-			2.5 (112)	
	Yes	-			17.7 (156)	
Material wealth index			<0.001			<0.001
	Lowest	2.2 (40)			1.9 (24)	
	Middle	7.2 (181)			2.2 (48)	
	Highest	36.4 (659)			10.9 (195)	
Total population		15.4 (13.4–17.7)			4.9 (4.0–6.0)	

The observed gradients between making PDVs and educational attainment, material wealth and nutritional status remained significant even after controlling for health insurance coverage (Table 2). In the general dentate population, having health insurance significantly increased the odds of making a yearly PDV (OR = 4.32; 95% CI = 3.04–6.14).

**Table 2:** Multi-level logistic model for preventive dental visits among the total dentate adult population (n=6,181).

Characteristics		Odds Ratio (95% Confidence Interval)	*p-value*
Age			
	per year increase	1.01 (1.00–1.02)	0.049
Ethnicity/race			
	Black African	1	
	Coloured	0.77 (0.42–1.40)	0.390
	Indian/Asian	1.60 (0.95–2.70)	0.077
	White	4.24 (2.54–7.09)	<0.001
Education			
	<High School	1	
	High school	2.36 (1.66–3.36)	<0.001
	>High school	2.12 (1.40–3.23)	<0.001
Material wealth index			
	Lowest	1	
	Middle	1.00 (0.59–1.72)	0.992
	Highest	1.89 (1.08–3.31)	0.026
Tobacco use			
	Never user	1	
	Ever snuff users	0.32 (0.10–1.04)	0.059
	Ever smokers	0.68 (0.48–0.96)	0.029
Nutritional status			
(food security)	Poorest	1	
	Middle	1.65 (1.15–2.37)	0.007
	Highest	2.43 (1.61–3.65)	<0.001
Privately insured			
	No	1	
	Yes	4.32 (3.04–6.14)	<0.001

There was a significant interaction between location and education (p = 0.010) and between race and education (p = 0.018). The racial and socio-economic differences in PDV observed in urban areas (Table 3) were not observed in the rural areas (Table 4).

**Table 3:** Final model predicting preventive dental visits for those residing in the urban areas.

Characteristics		Odds Ratio (95% Confidence Interval)	*p-value*
Race			
	Black African	1	
	Coloured	0.80 (0.42–1.53)	0.505
	Indian/Asian	1.35 (0.79–2.32)	0.276
	White	3.82 (2.21–6.60)	<0.001
Education			
	<High school	1	
	High school	2.84 (1.89–4.26)	<0.001
	>High school	2.70 (1.71–4.27)	<0.001
Material wealth index			
	Lowest	1	
	Middle	1.26 (0.49–3.21)	0.633
	Highest	2.79 (1.12–6.97)	0.028
Privately insured			
	No	1	
	Yes	5.26 (3.55–7.80)	<0.001

**Table 4:** Final model predicting preventive dental visits among those residing in the rural areas.

Characteristics		Odd Ratio (95% Confidence Interval)	*p-value*
Age			
	Per year increase	1.02 (1.00–1.03)	0.045
Nutritional status (food security)			
	Poorest	1	
	Middle	5.22 (2.12–12.84)	<0.001
	Highest	16.48 (6.78–40.05)	<0.001
Privately insured			
	No	1	
	Yes	4.64 (2.12–10.14)	<0.001
Tobacco use			
	Never	1	
	Ever snuff user	0.35 (0.09–1.30)	0.118
	Ever smoker	0.38 (0.14–1.02)	0.056

In the general dentate population, having health insurance accounted for the largest proportion (40.3%) of the White/non-White gap in the probability of reporting a PDV at least once a year (Table 5). The decomposition revealed that group differences in all of the measures of socio-economic position and health insurance enrollments included in the final overall model explained 55.9% (95% CI = 44.9–67.8) of the White/non-White gap in the probability of reporting making a PDV at least once a year (Table 5).

**Table 5:** Decomposition of the significant contributors to the White/non-White gap in PDVs.

Observed characteristics	Size of indirect effect relative to total effect (95% CI)	Predicted PDV among non-Whites * (compared to actual of 3.2%)
Health insurance	40.3% (31.2–49.3)	8.5%
Educational attainment	27.8% (20.9–34.8)	6.5%
Material wealth	27.6% (21.3–33.9)	6.2%
Nutritional status	10.3% (6.9–13.6)	4.2%
Location	7.4% (3.1–11.6)	3.9%
Domains combined	55.9% (44.9–67.8)	12.8%

* Displays predicted proportion of non-Whites that might make preventive dental visits (PDV) if they had the same distribution of observed characteristics as the Whites (the higher the figure, the smaller the White/non-White gap becomes).

## 3. Discussion

This study’s findings demonstrate that there is generally a very low level of routine PDVs in South Africa. Although a significantly higher rate of use was reported among those insured, slightly less than one in five insured South Africans routinely make PDVs. This study also showed that independent of health insurance status, individuals who are of low socio-economic position and are of other racial groups (non-Whites) as compared to Whites were significantly less likely to make PDVs. Furthermore, this study’s findings suggest that addressing racial differences in the socio-economic conditions measured and in health insurance ownership might result in a four-fold increase (*i.e.*, a predicted increase from 3.2% to 12.8%) in PDVs by non-Whites, thereby significantly reducing racial disparities in PDVs by about half (*i.e.*, from a gap of about 25% to 15%). Nevertheless, the fact that these social determinants also showed direct effects on PDVs further highlights the need to address these potential social determinants of oral health. It has indeed been suggested that the poor are likely to have to forgo food if they were to make a dental visit [20]. It therefore did not come as a surprise that those with the poorest nutritional status were less likely to make PDVs, particularly in the rural areas 

Conceivably, most of this study’s participants’ highest level of educational attainment is likely to have been determined several years before the current survey was carried out. Therefore, the study participants’ levels of educational attainment maybe related to parental socio-economic position, which in turn has been demonstrated to be associated with the level of attendance of dental services by adolescents [27]. Consequently, the fact that adults with lower levels of educational attainment were less likely to make PDVs, independent of their current material wealth is consistent with the life course theory [28]. The theory posits that early life events influence later adult health outcomes. In other words, an individual’s disease status or behaviour is a marker of the person’s past social position. Considering that behaviours related to preventive dental care are learnt from childhood, it was not surprising to find that health insurance status did not eliminate the observed disparities associated with educational status. Indeed, experience from the US and France also suggests that patients, particularly those with low educational attainment and low-income, may not necessarily take up the dental care opportunities offered under free, publicly-funded insurance programs [10,29]. 

The fact that health insurance coverage was not significantly associated with the proportion of those who reported dental problems in the preceding six months, and that particularly those in rural areas who had dental problems were less likely to make routine PDVs, suggest that the potential availability of funds for those with health insurance benefits or the availability of free primary dental care in the publicly-funded dental facilities in these areas was not enough to motivate making PDVs. Indeed, as in several low income countries, the public dental services in South Africa have remained extraction-driven [30] and opportunities for oral health promotion, such as providing preventive dental care, are missed in very busy and under-staffed publicly-funded clinics [13]. In general, about two-thirds of South African dental professionals work in private practices and there are much fewer private dental practices in the rural areas than in the urban areas of South Africa [25]. It is therefore conceivable that even if or when insurance funding is available for private dental care, fewer people would have access to preventive dental care in rural areas. 

Although the effect of having health insurance on making PDVs was not as strong among those living in rural areas as the effect on those in urban areas, the availability of health insurance was nevertheless a significant determinant of making PDVs among those in the rural areas. Considering that health insurance is employment-linked, it is possible that those with health insurance who live in rural areas may also be working in small towns, usually not too distant from their place of residence. Therefore, having employment-linked health insurance could provide them with the additional option of visiting private dental practices, which are more likely to be located in these small towns [25] and are also more likely than the overburdened publicly-funded dental facilities to provide preventive dental care. Nevertheless, considering that most of the effect of racial differences was not explained by differences in the location of the residences of the participants, this study highlights the need for future studies to investigate the direct role of characteristics of where people live on oral health care utilization.

Consistent with findings elsewhere [31], the fact that most of the White/non-White gap in PDVs was accounted for by racial differences in the distribution of health insurance enrollment suggests that the currently observed racial disparities in PDVs is mostly mediated by racial differences in the level of health insurance coverage. Moreover, consistent with previous observation that health insurance coverage may not eliminate inequities in service utilization [16], racial differences remained significant after controlling for health insurance status in this study. Although the decomposition analysis suggests that over half of the racial group differences in PDVs is accounted for by the factors considered in this study, about 44% of the White/non-White gap in PDVs remained unaccounted for by the observed characteristics reported in this study. This latter observation suggests that there are perhaps other patient-level or provider-level socio-economic, cultural or environmental factors [32] that have not been measured in this study but that can explain the observed racial disparities further. 

For instance, while the Coloured (those of mixed race) South Africans were more likely than black Africans to have health insurance, the proportion who reported PDVs was not significantly different. This could be related to cultural differences in beliefs on the cause of dental diseases and expectations regarding the benefits of PDVs. Indeed, a previous clinical study among health-insured South Africans demonstrated that differences in the level of education and a combination of the belief in the seriousness of the consequences of dental disease and the expected benefits of PDV significantly differentiated those who were prevention-oriented from those who were symptomatic-oriented in using dental services [33]. It is pertinent to note that although caries is highest among the Coloured population [34], a culture of the intentional removal of incisors as a form of dental modification is also predominantly practiced by Coloured South Africans [35]. It is therefore conceivable that Coloured South Africans, as compared to black Africans, particularly those of low socio-economic status, may be less likely to view the consequences of losing a tooth as being very serious and thus may have lower expectations regarding the benefits or value of PDVs. Furthermore, consistent with the findings of this study, smokers have been shown in other studies to be less likely to make dental visits [23]. Therefore, the fact that Coloured South Africans have the highest smoking rates [18] could also partly explain why fewer Coloured South Africans reported PDVs than would have been expected, based on their level of health insurance enrollment, as compared to the level of enrollment among black Africans. 

The findings from this study need to be interpreted within the limitations of the study’s design. Firstly, this was a cross-sectional study and therefore inferences on causality need to be made with caution, as there is no evidence of the temporal order of events. Nevertheless, it is likely that the socio-economic variables of race and education preceded dental care utilization. Secondly, the fact that we could not ascertain whether the health insurance coverage held by all those participants who had such coverage included a dental care benefit might explain why some of those who reported having a health insurance do not routinely make PDVs. Nevertheless, it is pertinent to note that most health insurance plans in South Africa include at least one visit to the dentist per year in their cover. However, more recently, some health insurance plans just make available a limited pool of funds that can be used for both out-of-hospital medical and dental care. Conceivably, dental care may be crowded out by medical care demands. Finally, the data presented is dated, and therefore may not represent the current situation. However, given that decreasing coverage of health insurance has been reported [4], it is less likely that the current situation would be significantly different from what is represented with regard to the role of health insurance. Moreover, this study presents information from the largest survey that could provide the kind of information presented here, given the low level of preventive dental care utilization. Furthermore, we have used the largest and most current nationally representative health survey that is publicly available in South Africa.

Despite this study’s limitations, this study has provided useful information that can inform policy debate and the design of more effective evidence-based interventions to reduce social disparities in preventive dental service utilization, and thus improve the oral health of South Africans. 

## 4. Conclusions

This study suggests that the extension of health insurance coverage to all South Africans may promote preventive dental visits and may reduce, but is unlikely to eliminate, the observed racial disparities in preventive dental service utilization. The study’s findings thus highlight the importance of the implementation of upstream oral health promotion strategies that address an improvement in the socio-economic conditions of people, in addition to implementing the proposed national health insurance scheme. 
